# A Facile Synthesis of *N*-H- and *N*-Substituted Acridine-1,8-diones under Sonic Condition

**DOI:** 10.1155/2013/930787

**Published:** 2013-12-31

**Authors:** S. Sudha, M. A. Pasha

**Affiliations:** Department of Studies in Chemistry, Bangalore University, Central College Campus, Palace Road, Bengaluru-560001, India

## Abstract

Synthesis of an assembly of structurally important *N*-H- and *N*-substituted acridine-1,8-diones by CAN (ceric ammonium nitrate) catalysed one-pot four-component reaction of electron-deficient and electron-rich aromatic aldehydes and aromatic amines or ammonium acetate and dimedone or cyclohexyl-1,3-diones at 26°C under sonic condition is reported. The method is clean and energy efficient as it uses a greener method and an eco-friendly catalyst.

## 1. Introduction

**Scheme 1:**
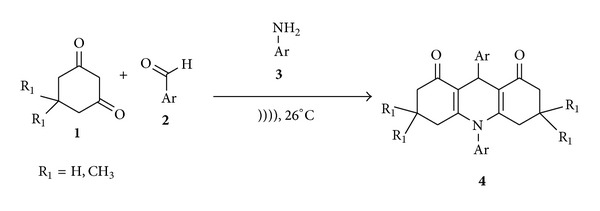
Synthesis of acridines under sonic condition.

**Scheme 2:**
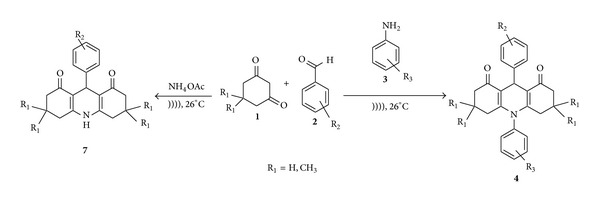
Synthesis of *N*-H-acridines (**7**) and *N*-substituted acridines (**4**).

Acridine and its derivatives are important structural motifs possessing antimalarial, antiviral, and antiallergic properties [1–3]; acridines act as potent drugs for antitumor activity both in vitro and in vivo against a range of murine and human tumors [4]. They are also found to act as fluorescent molecular probes for monitoring polymerization processes [5] and are used as *n*-type semiconductors and in the electroluminescent devices. Recently fluorinated acridones are reported to possess anticancer activity [6–9]. There are a few reports in the literature on the three-component Hantzsch-type condensation of aromatic aldehydes, anilines, and dimedone *via* traditional heating in organic solvents [10, 11], under microwave irradiation [12], and in ionic liquids [13]. The main drawbacks of these methods are the inability to synthesize profuse quantity of acridines using substituted anilines containing electron withdrawing groups [14]. Further, the reactions are carried out in refluxing organic solvents, which require higher temperature and longer hours for completion [10, 15] and unusual breaking of C–N bond takes place under certain reaction conditions as noticed in a few cases [16]. Hence, the exploration of a simple, efficient, and green method for the synthesis of acridines using electron-deficient amines and electron-deficient aldehydes is of current interest. In continuation with our work on one-pot multicomponent reactions under sonic condition [17–19], we, herein, report the synthesis of a series of acridines by a one-pot four-component reaction as shown in Schemes 1 and 2. To the best of our knowledge, the synthesis of acridines from fluorinated aromatic amines and heterocyclic amines using an inexpensive catalyst under sonic condition is not reported yet.

Sonic reactions are viewed as green and contemporary methods in synthetic organic chemistry. Sonochemical reactions are classified into three types based on their chemical effects induced by cavitation; they are homogeneous sonochemistry of liquids, heterogeneous sonochemistry of liquid-liquid or solid-liquid, and sonocatalysis (which overlaps the first two) and other mechanical effects. Study of chemical reactions under the mechanical effects sonic conditions is also called “false sonochemistry” which is also an important component of regular sonochemistry. In addition, the use of an inexpensive and versatile catalyst, CAN, in conjunction with ultrasound is considered to be economical and beneficiary in organic synthesis [20].

## 2. Methods

### 2.1. Materials and Instruments

All starting materials were commercial products and were used without further purification except liquid aldehydes and amines which were distilled before use. Melting points were measured on a Raaga make melting point apparatus. Nuclear magnetic resonance spectra were obtained on 400 MHz and 100 MHz Bruker AMX instruments in CDCl_3_ using TMS as a standard. ESI-Mass spectra were recorded using ESI-Q TOF instrument. All the reactions were carried out using SIDILU make sonic bath working at 35 kHz (constant frequency: 120 W) maintained at 26°C by circulating water without mechanical stirring. Yields refer to yield of the isolated products.

### 2.2. Typical Procedure for the Synthesis of Acridines

Dimedone/cyclohexa-1,3-dione (2 mmol), aromatic aldehyde (1 mmol), and substituted aniline (1 mmol) were taken in acetonitrile (0.5 mL) and mixed well, and to this CAN (5 mol%) was added. The reaction was subjected to ultrasonic irradiation in a bath working at 26°C (35 kHz). The course of the reaction was monitored on TLC (3 : 7: EtOAc : hexane). After completion of the reaction, water (10 mL) was added and the separated solid was filtered. The crude product was then subjected to silica gel column chromatography (3 : 7: EtOAc : hexane) to get the pure products. The same procedure was followed for the reactions carried out with ammonium acetate or 2-aminopyridine.

### 2.3. Spectral Data

 
**4a**: ^1^H NMR (400 MHz, CDCl_3_): **δ** 7.50–7.45 (m, 1H, ArH); 7.41–7.38 (m, 1H, ArH); 5.15 (s, 1H); 3.74 (s, 3H, OCH_3_); 2.44 (s, 2H); 2.25–2.15 (m, 4H); 2.14–1.96 (m, 1H); 0.98 (s, 3H, CH_3_); 0.93 (s, 3H, CH_3_); 0.84 (s, 3H, CH_3_).
 
^13^C NMR (100 MHz, CDCl_3_): **δ** 194.9 (C=O), 163.2 (Ph–C–F), 135–116 (Ph), 59.2 (OCH_3_), 49.8 (CH_2_), 40.1 (CH_2_), 32.6 (CH), 25.9 [C(CH_3_)_2_], ESI-MS: 507.2 (507.2). M.p: 265–267°C.
 
**4b**: ^1^H NMR (400 MHz, CDCl_3_): **δ** 7.39–7.37 (m, 1H, ArH); 7.28–7.20 (m, 4H, ArH); 7.19–1.17 (m, 1H, ArH); 7.05–7.02 (m, 2H, ArH); 6.99–6.83 (m, 1H, ArH); 5.6 (s, 1H); 2.25–2.17 (m, 4H); 2.06–2.03 (m, 2H); 1.83–1.62 (m, 4H); 0.99–0.88 (m, 12H, 4CH_3_).
 
^13^C NMR (100 MHz, CDCl_3_): **δ** 197.0 (C=O), 163.4 (Ph–C–F), 161.2 (Ph–C–F), 135–116 (Ph), 49.7 (CH_2_–CO), 40.2 (CH_2_–C), 32.5 (C, methine), 25.8 [C(CH_3_)_2_]. ESI-MS: 534.8 (495 + ^39^K). M.p: 240–242°C.
 
**4c**: ^1^H NMR (400 MHz, CDCl_3_): **δ** 7.39–7.38 (m, 1H, ArH); 7.37–7.28 (m, 1H, ArH); 7.26–7.20 (m, 1H, ArH); 7.18–7.05 (m, 2H, ArH); 7.02–6.83 (m, 1H, ArH); 5.7 (s, 1H); 2.25–2.17 (m, 2H); 2.06–1.99 (m, 2H); 1.66–1.58 (m, 6H); 0.95–0.88 (m, 12H, 4CH_3_).
 
^13^C NMR (100 MHz, CDCl_3_): **δ** 195.4, 163.4 (Ph–C–F), 149–125 (Ph and thiophene), 50.2 (CH_2_–CO), 40.2 (CH_2_–C), 32.8 (C, methine) 22.6 [C(CH_3_)_2_]. ESI-MS: 506 (483 + ^23^Na). M.p: 245–247°C.
 
**4d**: ^1^H NMR (400 MHz, CDCl_3_): **δ** 7.42–7.33 (m, 2H, ArH); 7.38–7.36 (m, 1H, ArH); 7.27–7.22 (m, 1H, ArH); 7.08–6.94 (m, 2H, ArH); 6.81–6.71 (m, 1H, ArH); 5.2 (s, 1H); 2.28–2.21 (m, 4H); 2.08–2.04 (d, *J* = 16, 2H); 1.90–1.86 (d, *J* = 16, 2H); 1.00 (s, 6H, 2CH_3_); 0.88 (s, 6H, 2CH_3_).
 
^13^C NMR (100 MHz, CDCl_3_): **δ** 195.6 (C=O), 153.4 (Ph–C–F), 135–115 (Ph), 49.9 (CH_2_–CO), 41.7 (CH_2_–C), 32.1 (C, methine), 27.4 [C(CH_3_)_2_]. ESI-MS: 512.1 (511.1 + ^1^H). M.p: 250–252°C.
 
**4e**: ^1^H NMR (400 MHz, CDCl_3_): **δ** 7.56–7.41 (m, 2H, ArH); 7.41–7.39 (m, 1H, ArH); 7.31–7.20 (m, 5H, ArH); 7.17–7.06 (m, 1H, ArH); 5.20 (s, 1H); 2.19–2.13 (m, 4H); 1.62–1.57 (m, 4H); 0.93 (s, 6H, 2CH_3_); 0.83 (s, 6H, 2CH_3_).
 
^13^C NMR (100 MHz, CDCl_3_): **δ** 197.0 (C=O), 161.2 (Ph–C–F), 135–115 (Ph), 49.0 (CH_2_–CO), 40.2 (CH_2_–C(CH_3_)_2_), 32.5 (C, methine), 25.8 [C(CH_3_)_2_]. ESI-MS: 500.1 (477.1 + ^23^Na). M.p: 260–262°C.
 
**4f**: ^1^H NMR (400 MHz, CDCl_3_): **δ** 7.4–6.7 (m, 9H, ArH); 5.2 (s, 1H); 2.3–2.26 (m, 4H); 2.21–2.17 (d, *J* = 16, 2H); 2.08–2.04 (d, *J* = 16, 2H); 1.6 (s, 3H); 1.07 (s, 6H, 2CH_3_); 0.87 (s, 6H, 2CH_3_).
 
^13^C NMR (100 MHz, CDCl_3_): **δ** = 197.0 (C=O), 161.2 (Ph–C–F), 150.6 (Ph–C–OH), 135–115 (Ph), 50.0 (CH_2_–CO), 40.2 (CH_2_–C), 32.5 (C, methine), 25.8 [C(CH_3_)_2_]. ESI-MS: 516.1 (493.1 + ^23^Na). M.p: 253–255°C.
 
**8a**: ^1^H NMR (400 MHz, CDCl_3_): **δ** 7.56–7.41 (m, 1H, ArH); 7.41–7.39 (m, 2H, ArH); 7.31–7.21 (m, 4H, ArH); 7.20–7.08 (m, 2H, ArH); 5.2 (s, 1H); 2.19–2.13 (m, 4H); 1.62–1.57 (m, 4H); 0.93 (s, 6H, 2CH_3_); 0.83 (s, 6H, 2CH_3_). ESI-MS: 426.2 (449.3 + ^23^Na). M.p: 258–263°C.
 
**8b**: ^1^H NMR (400 MHz, CDCl_3_): **δ** 7.13–6.63 (m, 8H, ArH); 5.2 (s, 1H); 3.50–3.27 (m, 2H); 2.72–2.54 (m, 2H); 2.26–2.08 (m, 2H); 1.06 (s, 6H, 2CH_3_). ESI-MS: 398.2 (399.2 + ^1^H). M.p: 263–267°C.

## 3. Results and Discussion

To begin with, we planned to work with highly electron deficient 2-chloro-4-fluoroaniline (1 mmol), dimedone (2 mmol), and an electron deficient 4-fluorobenzaldehyde (1 mmol) in 3–5 mL acetonitrile as a solvent. We studied the reaction using various Lewis acid catalysts such as ZnCl_2_, ZnBr_2_, SnCl_4_, AlCl_3_, CuCl, and CAN under sonic condition (26°C, 35 kHz) and found that CAN (5 mole%) catalysed the reaction effectively and gave very high yield (90%, 1 h) of the product under sonic condition, and with other catalysts the yield was below 40% after 2 h.

**Scheme 3:**
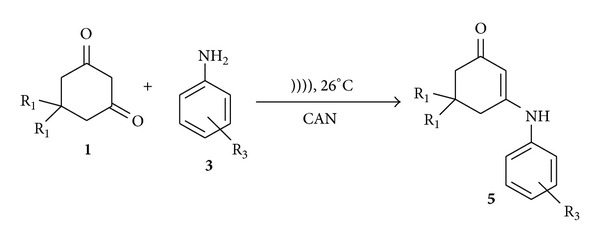
Formation of *β*-enaminones.

To understand the effect of ultrasound on the present reaction, we carried out a comparative study on the CAN catalysed reaction under sonic and silent condition. Under silent condition, the reaction was carried out using dimedone (2 mmol), 2-chloro-4-fluoroaniline (1 mmol), and 4-fluorobenzaldehyde (1 mmol) in acetonitrile (3–5 mL) as a solvent at 70°C for 4 h, and we observed the formation of acridine-1,8-dione in 50% yield (Table 1, entry 3). This is because formation of *β*-enaminone (Scheme 2) under silent condition from electron-deficient aniline and an aldehyde is generally difficult; on sonication (26°C, 35 kHz) the yield was 90% after 1 h (entry 3) (Scheme 3).

**Scheme 4:**
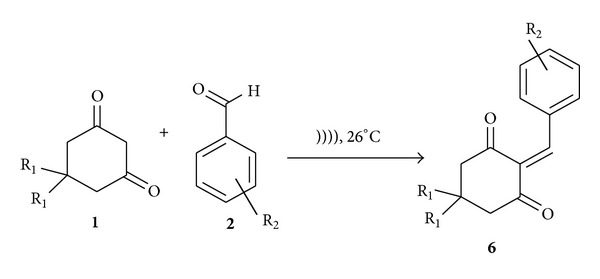
Formation of Knoevenagel adduct.

**Scheme 5:**
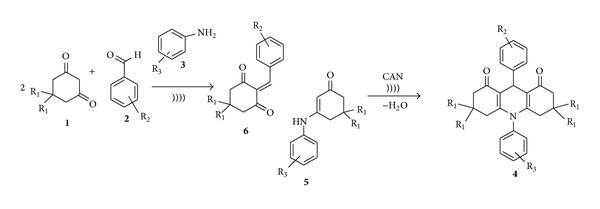
Formation of *N*-substituted acridines.

In order to understand the role of ultrasound and the catalyst we decided to study the mechanism of formation of acridines in detail. From the literature, it is clear that formation of acridines involves the condensation of *β*-enaminone (**5**), with the Knoevenagel adduct **6** derived from the dimedone or cyclohexan-1,3-dione and an aromatic aldehyde as shown in Scheme 4, followed by the cyclization of intermediates **A** and **B** followed by the removal of a molecule of water to give the product as shown in Scheme 5. We feel that the catalyst may activate the intermediates **6**, **A**, and **B** to give product **4**. Although the formation of intermediates **5** and **6** is a must, isolation of these intermediates is not a necessary step under sonic condition, mixing all the starting materials and CAN, and subjecting the mixture to sonication in acetonitrile 26°C will give the products in very high yields as shown in Table 1 (entry 3).

**Scheme 6:**
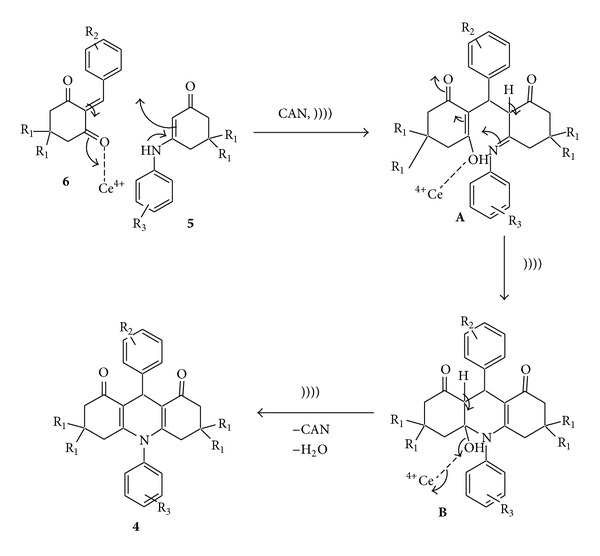
A plausible mechanism for the formation of acridines from **5 **and **6**.

After optimizing the reaction conditions, we applied the optimized condition to a range of other aromatic aldehydes and aromatic amines, ammonium acetate, and a heterocyclic amine; the results of this study are presented in Table 2. The reaction using ammonium acetate was found to be faster than the reaction with aromatic amines and aniline under sonic condition. Electron releasing and electron withdrawing groups on aldehydes and amines did not show much effect on the rates and yield of the reaction under sonic condition. To study the efficacy of the developed method, we extended the work to heterocyclic aldehyde: thiophene-2-aldehdye (Table 2, entry 3) and heterocyclic amine: 2-aminopyridine (Table 2, entries 11 and 12), and found to give the respective products in very high yield (Scheme 6).

### 3.1. Effect of Sonication

The noteworthy qualities of the sonication are improved reaction rates and formation of pure products in high yields. Additionally, sonication allows benefits like easier manipulation, improved energy conservation, and low waste generation as compared to the traditional methods; in recent years, the sonochemical energy delivery has been used as an excellent alternative to thermal energy in promoting organic reactions. The origin of the energy lies in the “cavitation” phenomenon which involves formation, growth, and collapse of several millions of tiny vapour bubbles in the liquid medium [21, 22]. This effect is mainly responsible for generating strong convection in the liquid media through several mechanisms such as microstreaming, microturbulence, shock waves, and microjets. The fast imploding bubbles generate extremely high temperatures of the order of 5000°C and very high pressures of about 1000 atm within the cavity. In such microreactors sound energy is transformed into the chemical energy [23]. The possible nuclei for the “cavitation” are also the gas pockets trapped in the walls and crevices of the solid reagents, reactants, and vessel walls, or they could be small bubbles already present in the reaction medium. Thus the collapsing bubbles generate localized “hot spots” and the reactant molecules enter into the cavities where dissolution of reactants can take place; molecular fragmentation can also take place and can produce highly reactive intermediates and species. Cavitation is responsible for the chemical effects including the acceleration of the rates of the reactions through the heat and the mass transformations in homogeneous solutions [24].

We have applied the effective combination of suitable solvents and ultrasound in the synthesis of heterocyclic and other important compounds through one-pot multicomponent reaction strategies under sonic condition earlier [18, 19]. Therein, we have explained the effectiveness of these combinations in improving the reaction parameters and conserving the energy. We have now applied this strategy in the synthesis of *N*-H- and *N*-substituted acridines by a one-pot four-component reaction under sonic condition as shown in Scheme 4.

## 4. Conclusions

To conclude, we have developed a general, practical, and high yielding procedure to construct different *N*-H- and *N*-substituted acridines from electron-deficient as well as electron-rich aromatic aldehydes and aromatic amines or ammonium acetate and dimedone or cyclohexyl-1,3-diones at 26°C under sonic condition (35 kHz). High yields, shorter reaction durations, and mild reaction conditions are the added advantages of our energy efficient method.

## Supplementary Material

Synthesis of an assembly of structurally important N-H and N-substituted-acridine-1,8-diones by CAN catalysed one-pot four-component reaction of electron-deficient and electron-rich aromatic aldehydes and aromatic amines or ammonium acetate and dimedone or cyclohexyl-1,3-diones at 26°C under sonic condition is reported. The method is clean and energy efficient as it uses a greener method and an eco-friendly catalyst. The products have been characterized by 1HNMR, 13CNMR and HRMS analysis.

## Figures and Tables

**Table 1:** Comparison between CAN catalysed silent and sonic reactions.

Entry	Reactions	Silent condition^a^		Sonic condition^b^	
		Time	Yield^c^	Time	Yield^c^
1	Dimedone (1 mmol) and 2-chloro-4-fluoroaniline (1 mmol) to form **5**	45 min	75%	20 min	95%
2	Reaction between intermediates **5** and **6 **to give product** 4**	4 h	50%	1 h	90%
3	Mixing all the reactants (**1**, **2**, and **3**)	5 h	50%	1 h	90%

^a^In acetonitrile (3–5 mL) at 70°C. ^b^In a sonic bath at 26°C (35 kHz). ^c^Isolated yields.

**Table 2:** A small library of acridines synthesized under sonic condition^a^.

Entry	(**1**)	(**2**)	Amines (**3**)	Product	Yield^b^ %	Time (min)
1	R_1_ = CH_3_	4-Methoxy benzaldehyde	4-Chloro, 2-fluoroaniline (**3a**)	**4a** ^†^	90	40
2	R_1_ = CH_3_	2-Fluoro benzaldehyde	(**3a**)	**4b** ^†^	95	45
3	R_1_ = CH_3_	Thiophene-2-aldehdye	(**3a**)	**4c** ^†^	95	45
4	R_1_ = CH_3_	4-Chloro benzaldehyde	(**3a**)	**4d** ^†^	93	45
5	R_1_ = CH_3_	Benzaldehyde	(**3a**)	**4e** ^†^	95	45
6	R_1_ = CH_3_	4-Hydroxy benzaldehyde	(**3a**)	**4f** ^†^	90	40
7	R_1_ = CH_3_	Benzaldehyde	Aniline (**3b**)	**4g** ^¥^	95	40
8	R_1_ = CH_3_	4-Chloro benzaldehyde	(**3b**)	**4h** ^¥^	93	40
9	R_1_ = H	4-Chloro benzaldehyde	(**3b**)	**4j** ^¥^	90	40
10	R_1_ = H	Benzaldehyde	NH_4_Ac	**7a** ^¥^	90	20
11	Dimedone	4-Chloro benzaldehyde	NH_4_Ac	**7b** ^¥^	95	20
12	R_1_ = CH_3_	Benzaldehyde	2-Aminopyridine	**8a** ^†^	90	50
13	Dimedone and cyclohexa-1,3-dione	Benzaldehyde	2-Aminopyridine	**8b** ^†^	85	50

^a^Reaction condition as mentioned in general procedure. ^b^Isolated yields. ^†^Novel compounds. ^¥^Known compounds were characterized by comparison of their physical data and on TLC with the authentic samples.

## References

[1] Girault S, Grellier P, Berecibar A (2000). Antimalarial, antitrypanosomal, and antileishmanial activities and cytotoxicity of bis(9-amino-6-chloro-2-methoxyacridines): influence of the linker. *Journal of Medicinal Chemistry*.

[2] Kelly JX, Smilkstein MJ, Brun R (2009). Discovery of dual function acridones as a new antimalarial chemotype. *Nature*.

[3] Thuli U, Testa B (1994). Screening of unsubstituted cyclic compounds as inhibitors of monoamine oxidases. *Biochemical Pharmacology*.

[4] Demeunynck M, Charmantray F, Martelli A (2001). Interest of acridine derivatives in the anticancer chemotherapy. *Current Pharmaceutical Design*.

[5] Popielarz R, Hu S, Neckers DC (1997). Applicability of decahydroacridine-1,8-dione derivatives as fluorescent probes for monitoring of polymerization processes. *Journal of Photochemistry and Photobiology A*.

[6] Papagni A, Del Buttero P, Moret M, Sassella A, Miozzo L, Ridolfi G (2003). Synthesis and properties of some derivatives of 1,2,3, 4-tetrafluoroacridine for solid state emitting systems. *Chemistry of Materials*.

[7] Giovanella U, Botta C, Papagni A, Tubino R, Miozzo L (2005). Electroluminescence from two fluorinated organic emitters embedded in polyvinylcarbazole. *Applied Physics Letters*.

[8] Miozzo L, Papagni A, Cerminara M, Meinardi F, Tubino R, Botta C (2004). Sensitised green emission in an electrically active polymer doped with a fluorinated acridine. *Chemical Physics Letters*.

[9] Fadeyi OO, Adamson ST, Myles EL, Okoro CO (2008). Novel fluorinated acridone derivatives—part 1: synthesis and evaluation as potential anticancer agents. *Bioorganic and Medicinal Chemistry Letters*.

[10] Das B, Thirupathi P, Mahender I, Reddy VS, Rao YK (2006). Amberlyst-15: an efficient reusable heterogeneous catalyst for the synthesis of 1,8-dioxo-octahydroxanthenes and 1,8-dioxo-decahydroacridines. *Journal of Molecular Catalysis A*.

[11] Martin N, Quinteiro M, Seoane C (1995). Synthesis of conformational study of acridine derivatives related to 1,4-dihydropyridines. *Journal of Heterocyclic Chemistry*.

[12] Tu S-J, Miao C-B, Gao Y, Feng Y-J, Feng J-C (2002). Microwave-prompted reaction of cinnamonitrile derivatives with 5,5-dimethyl-1,3-cyclohexanedione. *Chinese Journal of Chemistry*.

[13] Shi D-Q, Ni S-N, Fang-Yang F-Y (2008). An efficient synthesis of polyhydroacridine derivatives by the three-component reaction of aldehydes, amines and dimedone in ionic liquid. *Journal of Heterocyclic Chemistry*.

[14] Jin T-S, Zhang J-S, Guo T-T, Wang A-Q, Li T-S (2004). One-pot clean synthesis of 1,8-dioxo-decahydroacridines catalyzed by *p*-dodecylbenezenesulfonic acid in aqueous media. *Synthesis*.

[15] Kidwai M, Bhatnagar D (2010). Ceric ammonium nitrate (CAN) catalyzed synthesis of N-substituted decahydroacridine-1,8-diones in PEG. *Tetrahedron Letters*.

[16] Muscia GC, Buldain GY, Asís SE (2009). Only acridine derivative from Hantzsch-type one-pot three-component reactions. *Monatshefte fur Chemie*.

[17] Datta B, Pasha MA (2011). Cavitational chemistry: a mild and efficient multi-component synthesis of amidoalkyl-2-naphthols using reusable silica chloride as catalyst under sonic conditions. *Ultrasonics Sonochemistry*.

[18] Sudha S, Pasha MA (2012). Ultrasound assisted synthesis of tetrahydrobenzo[c]xanthene-11-ones using CAN as catalyst. *Ultrasonics Sonochemistry*.

[19] Datta B, Pasha MA (2012). Glycine catalyzed convenient synthesis of 2-amino-4*H*-chromenes in aqueous medium under sonic condition. *Ultrasonics Sonochemistry*.

[20] Mason TJ (1997). Ultrasound in synthetic organic chemistry. *Chemical Society Reviews*.

[21] Zang H, Zhang Y, Zang Y, Cheng B-W (2010). An efficient ultrasound-promoted method for the one-pot synthesis of 7,10,11,12-tetrahydrobenzo[c]acridin-8(9H)-one derivatives. *Ultrasonics Sonochemistry*.

[22] Heravi MRP (2009). An efficient synthesis of quinolines derivatives promoted by a room temperature ionic liquid at ambient conditions under ultrasound irradiation via the tandem addition/annulation reaction of *o*-aminoaryl ketones with *α*-methylene ketones. *Ultrasonics Sonochemistry*.

[23] Prasad K, Pinjari DV, Pandit AB, Mhaske ST (2010). Phase transformation of nanostructured titanium dioxide from anatase-to-rutile via combined ultrasound assisted sol-gel technique. *Ultrasonics Sonochemistry*.

[24] Petrier C, Luche JL (1998). *Synthetic Organic Sonochemistry*.

