# Supplementary material for: A Facile Synthesis of N-H- and N-Substituted Acridine-1,8-diones under Sonic Condition
Source: ScientificWorldJournal. 2013 Dec 31;2013:930787. doi: 10.1155/2013/930787 (PMC3899711; doi:10.1155/2013/930787)
Supplement: Supplementary file 1 — Synthesis of an assembly of structurally important N-H and N-substituted-acridine-1,8-diones by CAN catalysed one-pot four-component reaction of electron-deficient and electron-rich aromatic aldehydes and aromatic amines or ammonium acetate and dimedone or cyclohexyl-1,3-diones at 26°C under sonic condition is reported. The method is clean and energy efficient as it uses a greener method and an eco-friendly catalyst. The products have been characterized by 1HNMR, 13CNMR and HRMS analysis. [file 930787.f1.doc]

**CAN catalysed, facile, one-pot four-component synthesis of some structurally important acridin-1,8-diones under sonic condition**

**S. Sudha** and **M. A. Pasha** *

Department of Studies in Chemistry, Central College Campus, Palace Road,

Bangalore University, Bengaluru- 560001, INDIA

*Corresponding author. Email: M. A. Pasha- [m_af_pasha@ymail.com](mailto:m_af_pasha@ymail.com)

**6a**: 10-(2′-chloro-4′-fluorophenyl)-9-(4″-methoxyphenyl)-3,3,6,6-tetramethyl-3,4,6,7,9,10-hexahydroacridin-1,8-(2*H*,5*H*)-dione


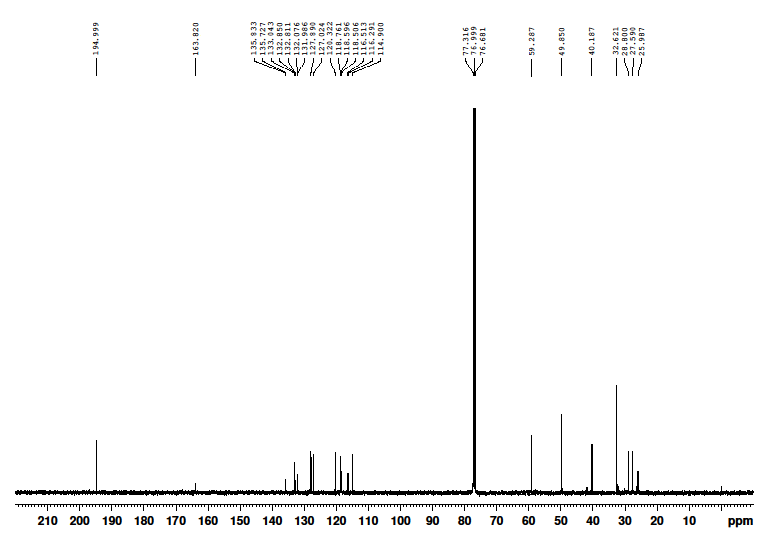


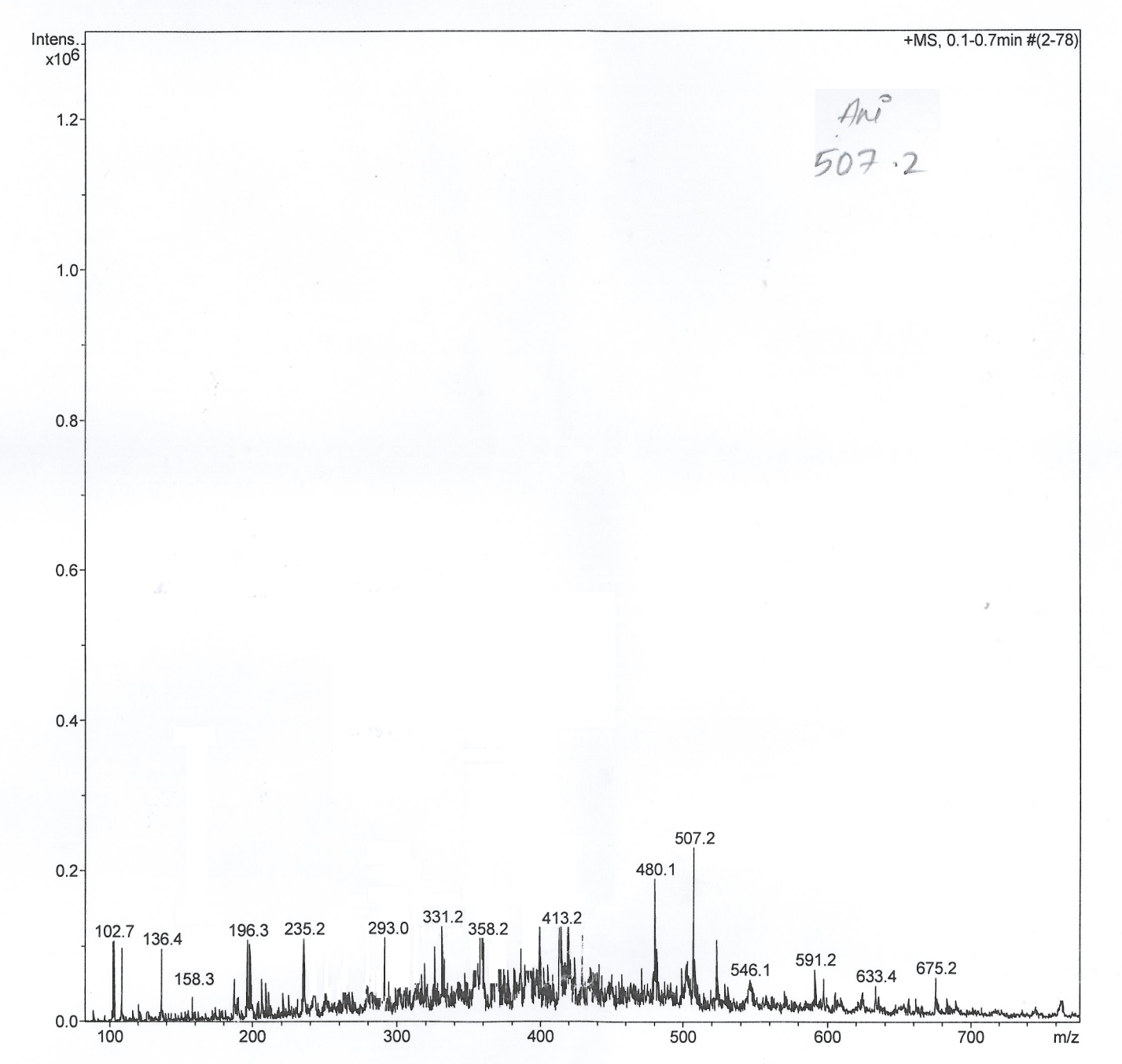


**6b:** 10-(2′-chloro-4′-fluorophenyl)-9-(2″-fluorophenyl)-3,3,6,6-tetramethyl-3,4,6,7,9,10-hexahydroacridin-1,8-(2*H*,5*H*)-dione


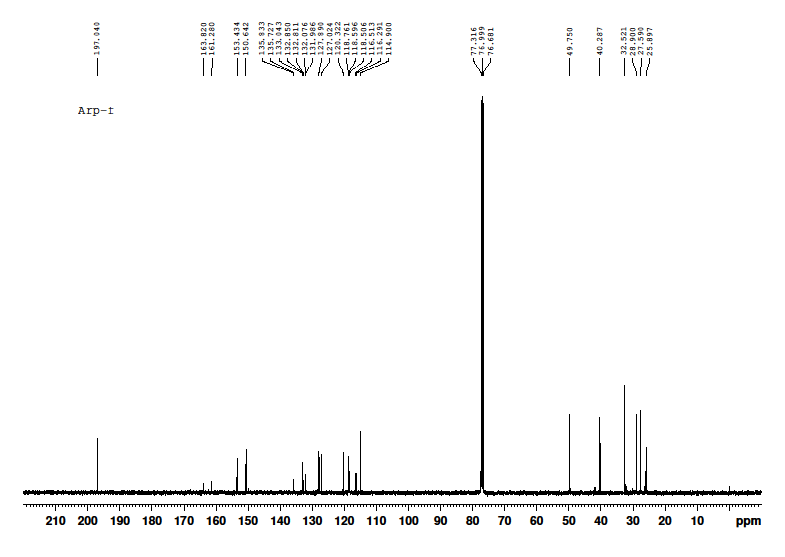


## ESI-MS: 534.8 (495 + ^39^K).

##
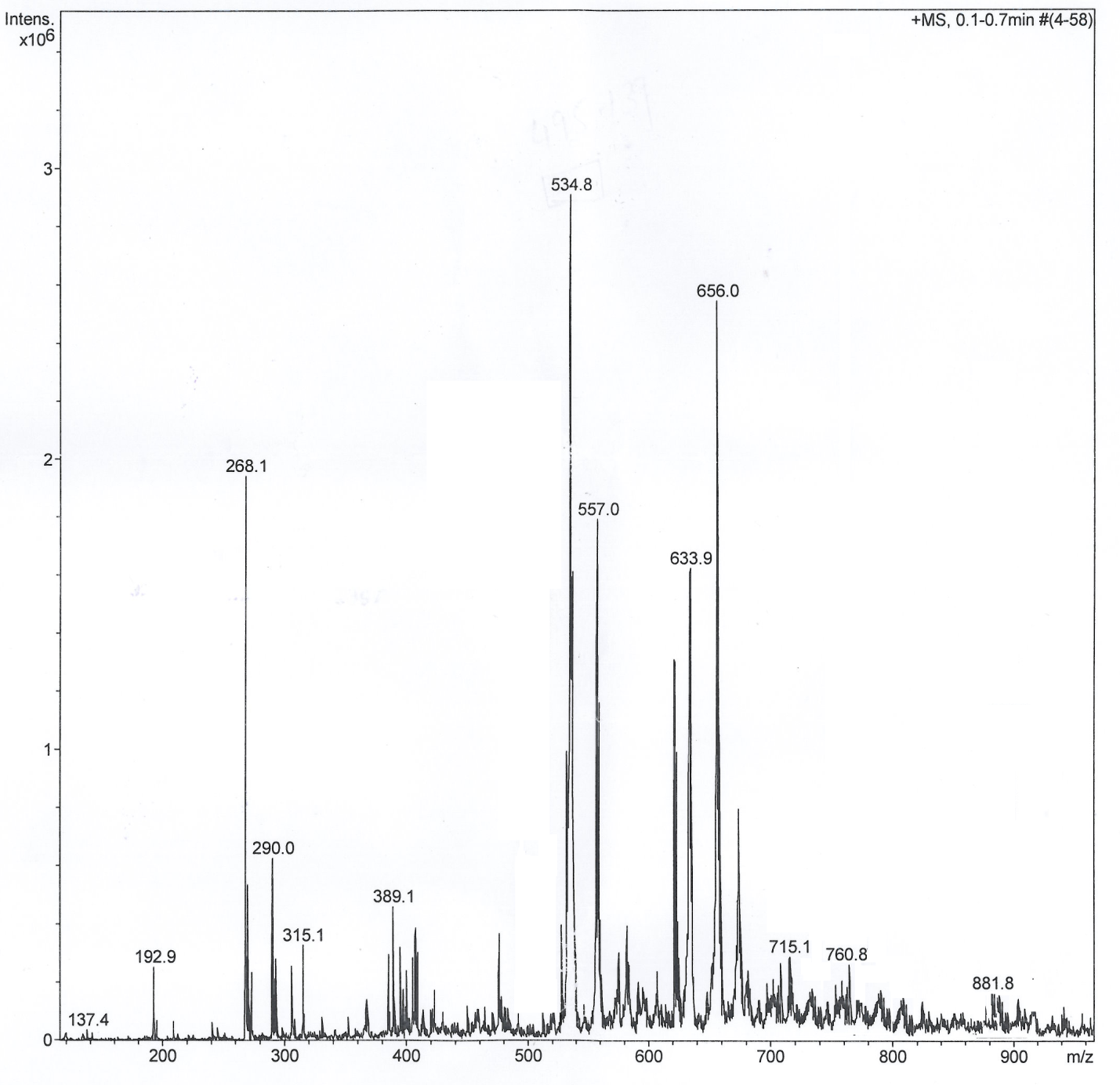


**6c**: 10-(2′-chloro-4′-fluorophenyl)-3,3,6,6-tetramethyl-9-(thiophen-2-yl)-3,4,6,7,9,10-hexahydroacridine-1,8-(2*H*,5*H*)-dione


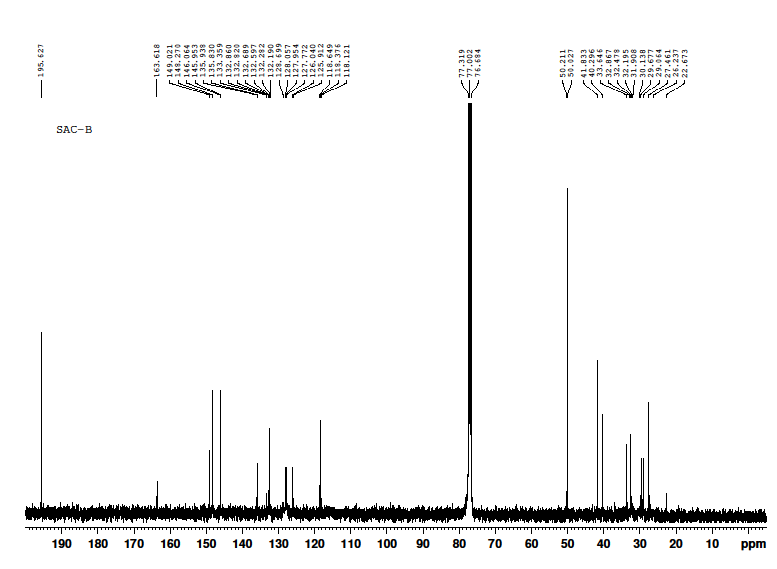


ESI-MS: 506 (483 + ^23^Na)


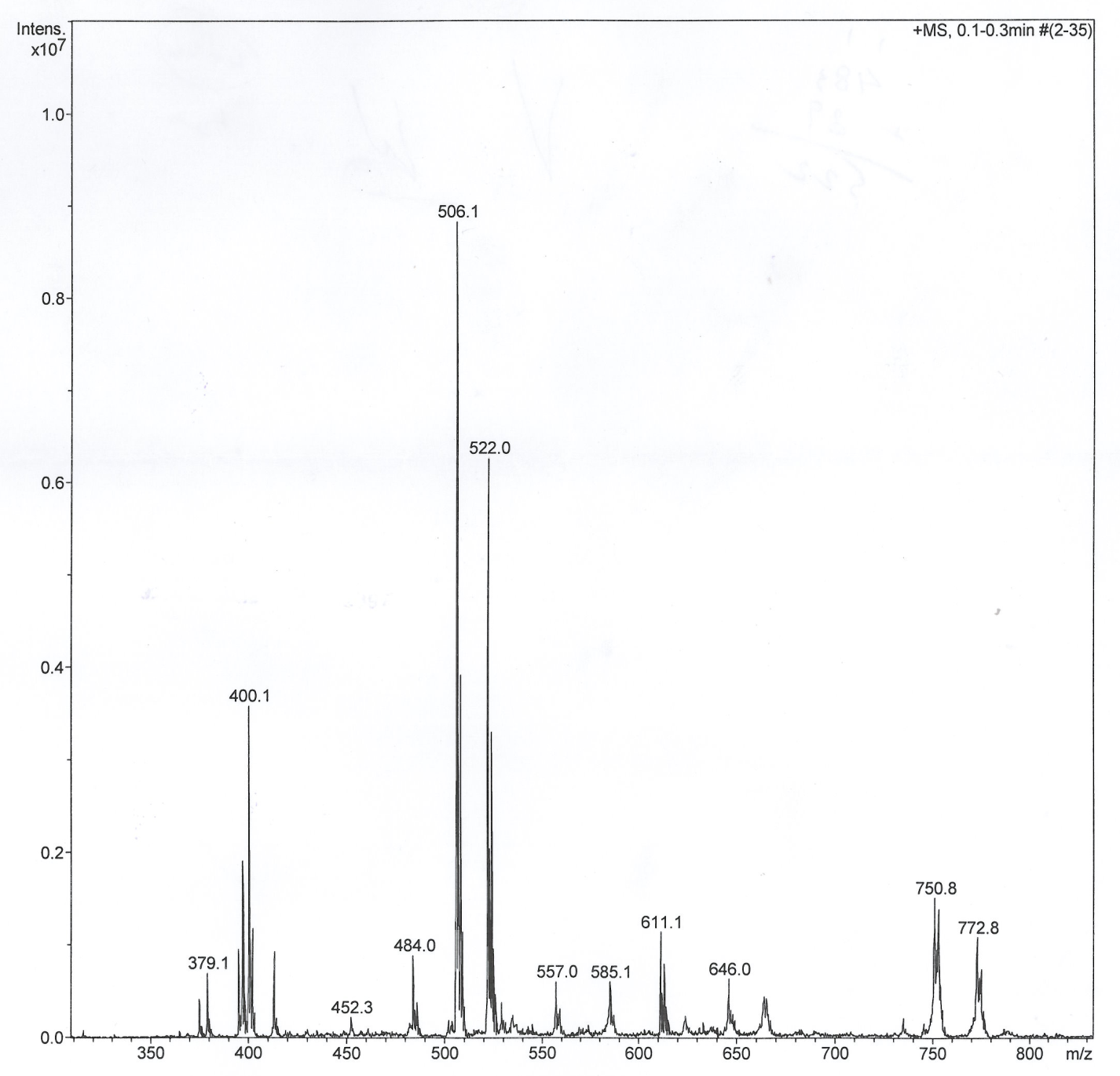


**6d**: 10-(2′-chloro-4′-fluorophenyl)-9-(4″-chlorophenyl)-3,3,6,6-tetramethyl-3,4,6,7,9,10-hexahydroacridin-1,8-(2*H*,5*H*)-dione


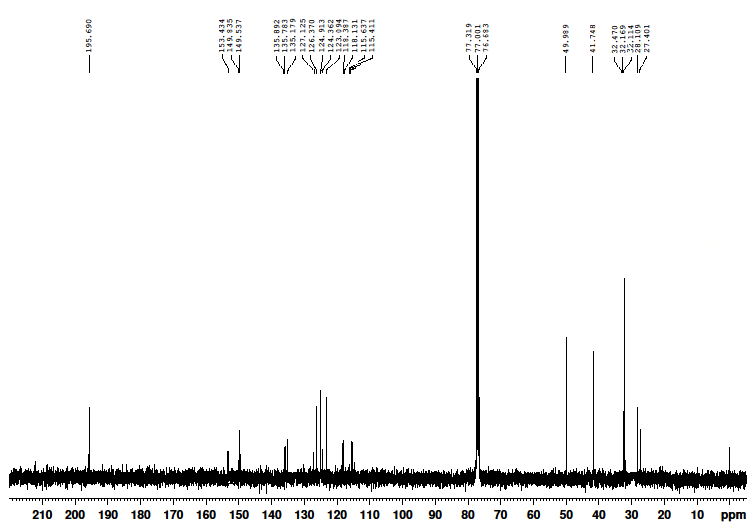


## ESI-MS: 512.1 (511.1 + ^1^H)


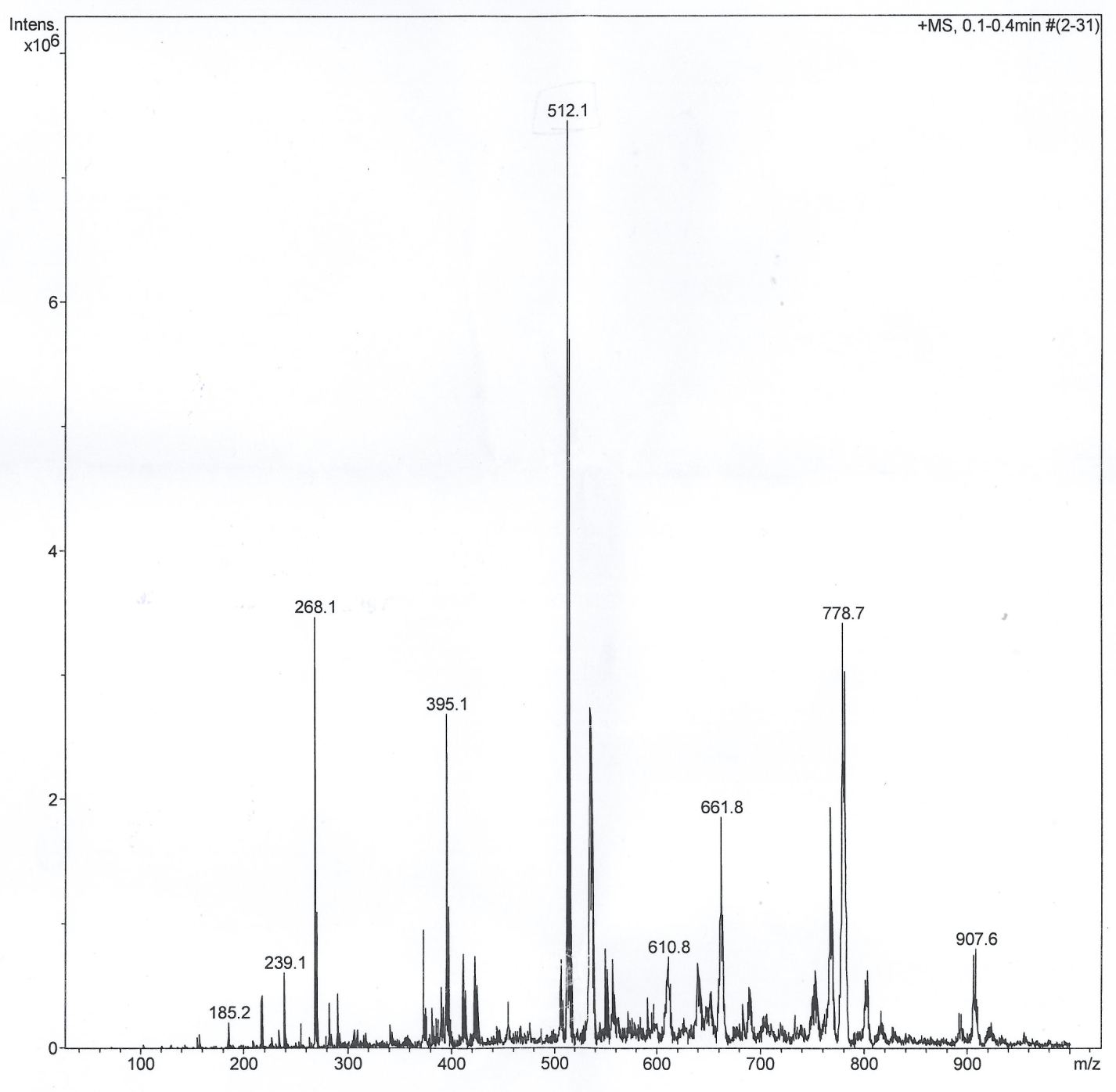


**6e**: 10-(2′-Chloro-4′-fluoro-phenyl)-3,3,6,6-tetramethyl-9-phenyl-3,4,6,7,9,10-hexahydroacridin -1,8-(2*H*,5*H*)-dione


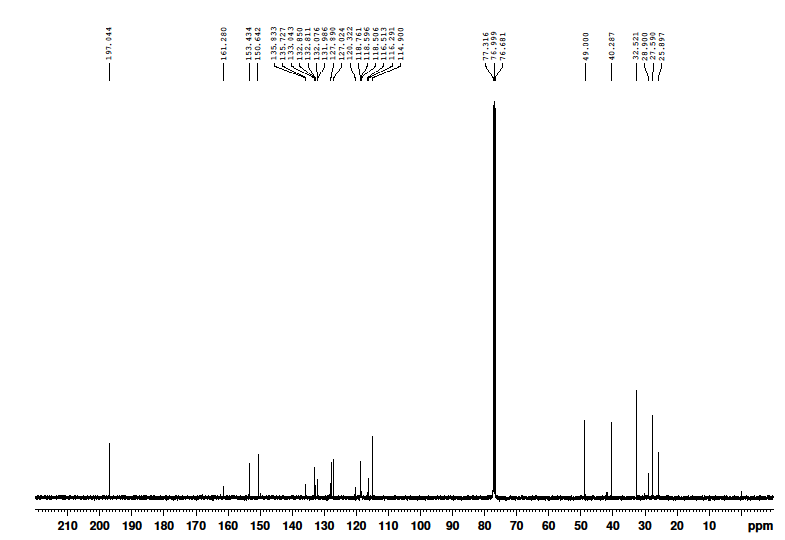


ESI-MS: 500.1 (477.1 + ^23^Na)


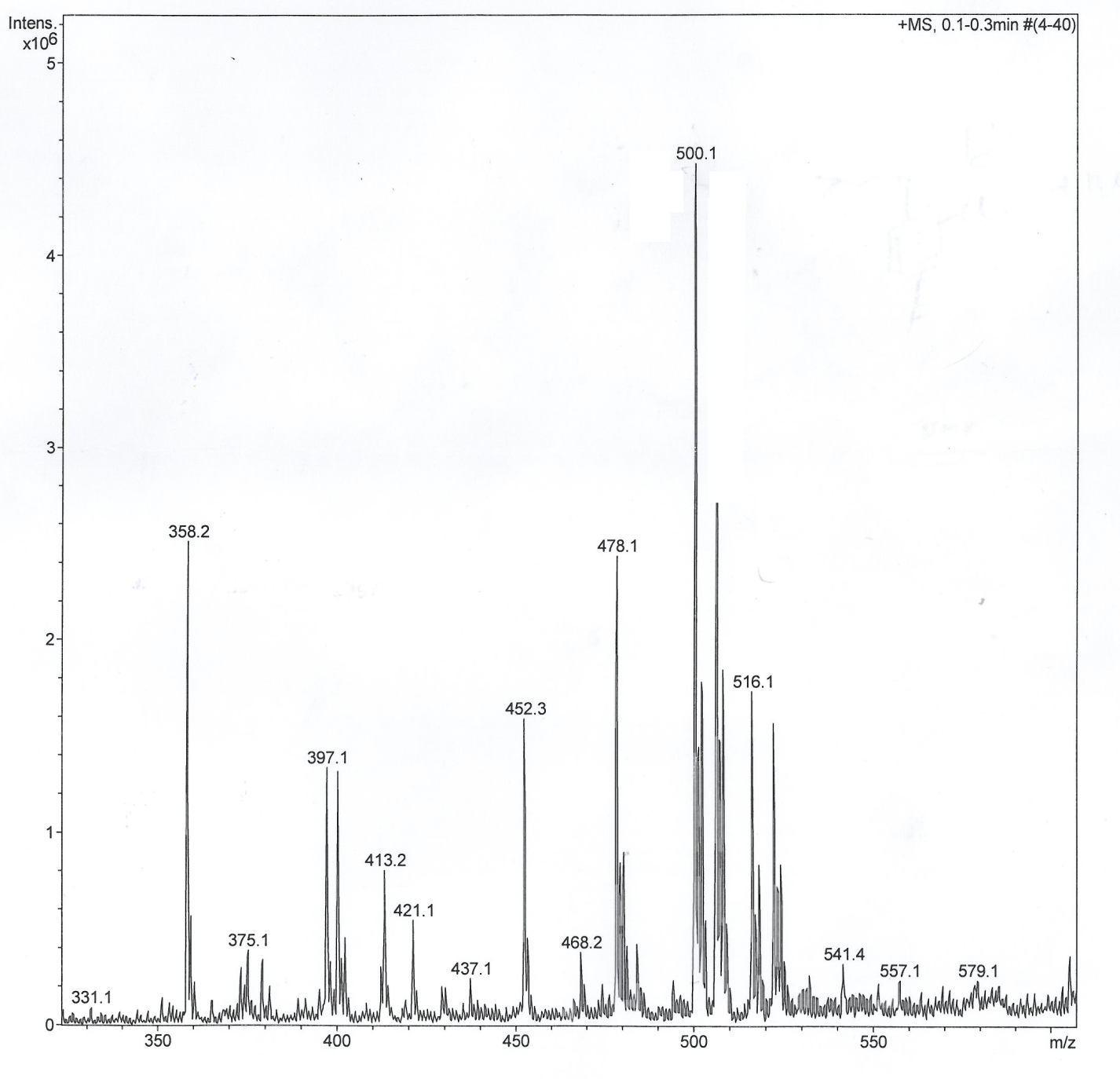


**6f**: 10-(2′-chloro-4′-fluorophenyl)-9-(4″-hydroxyphenyl)-3,3,6,6-tetramethyl-3,4,6,7,9,10-hexahydroacridin-1,8-(2*H*,5*H*)-dione


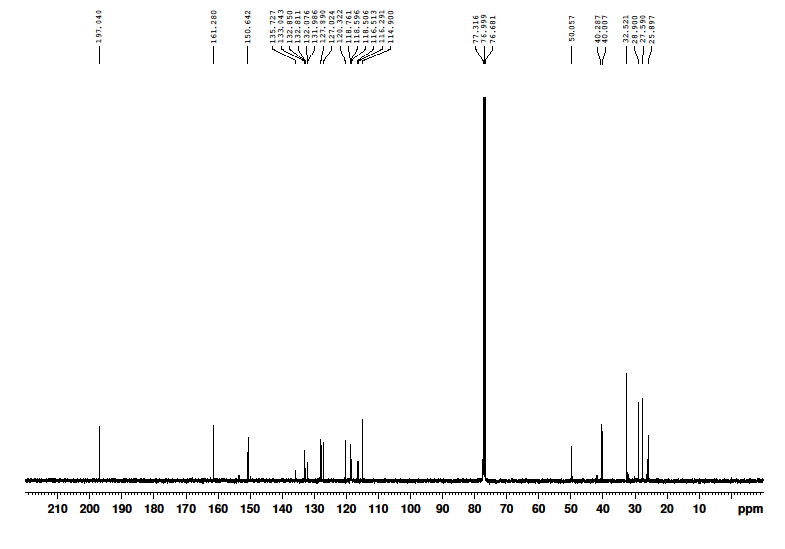


ESI-MS: 516.1 (493.1 + ^23^Na)


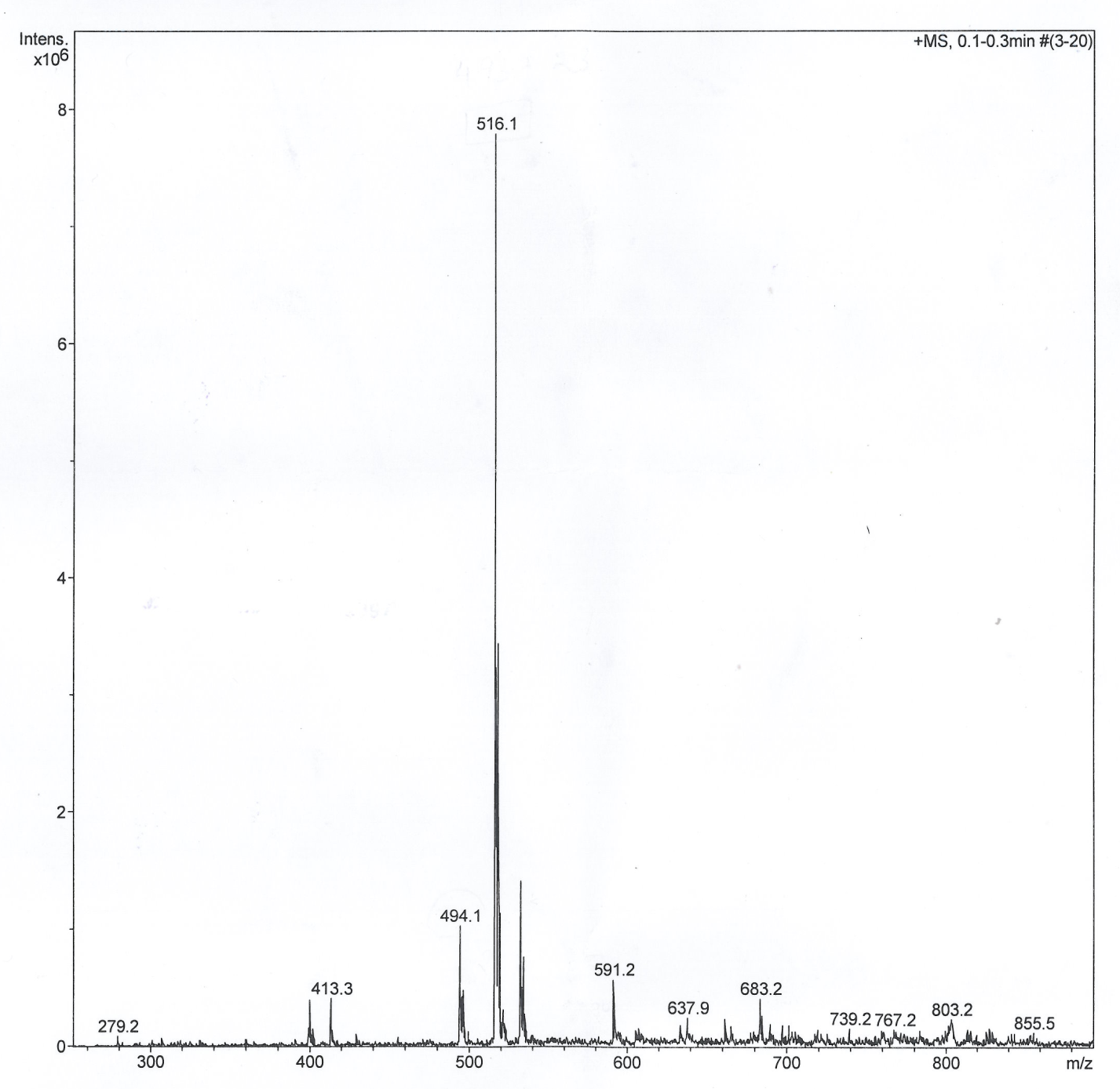


**8a**: 3,4,6,7-tetramethyl-9-(phenyl)-3,3,6,6-tetramethyl-10-(pyridine-2-yl)-acridin-1,8-(2*H*,5*H*,9*H*,10*H*)-dione


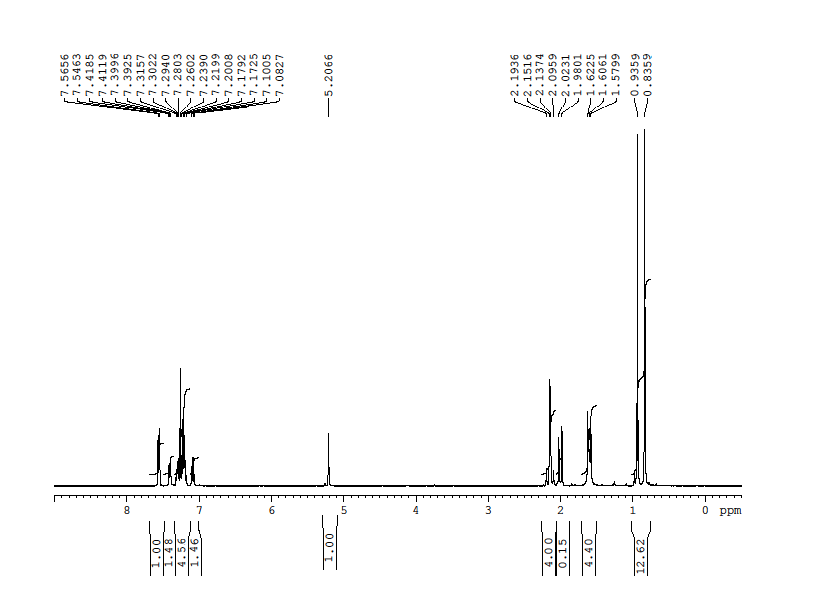


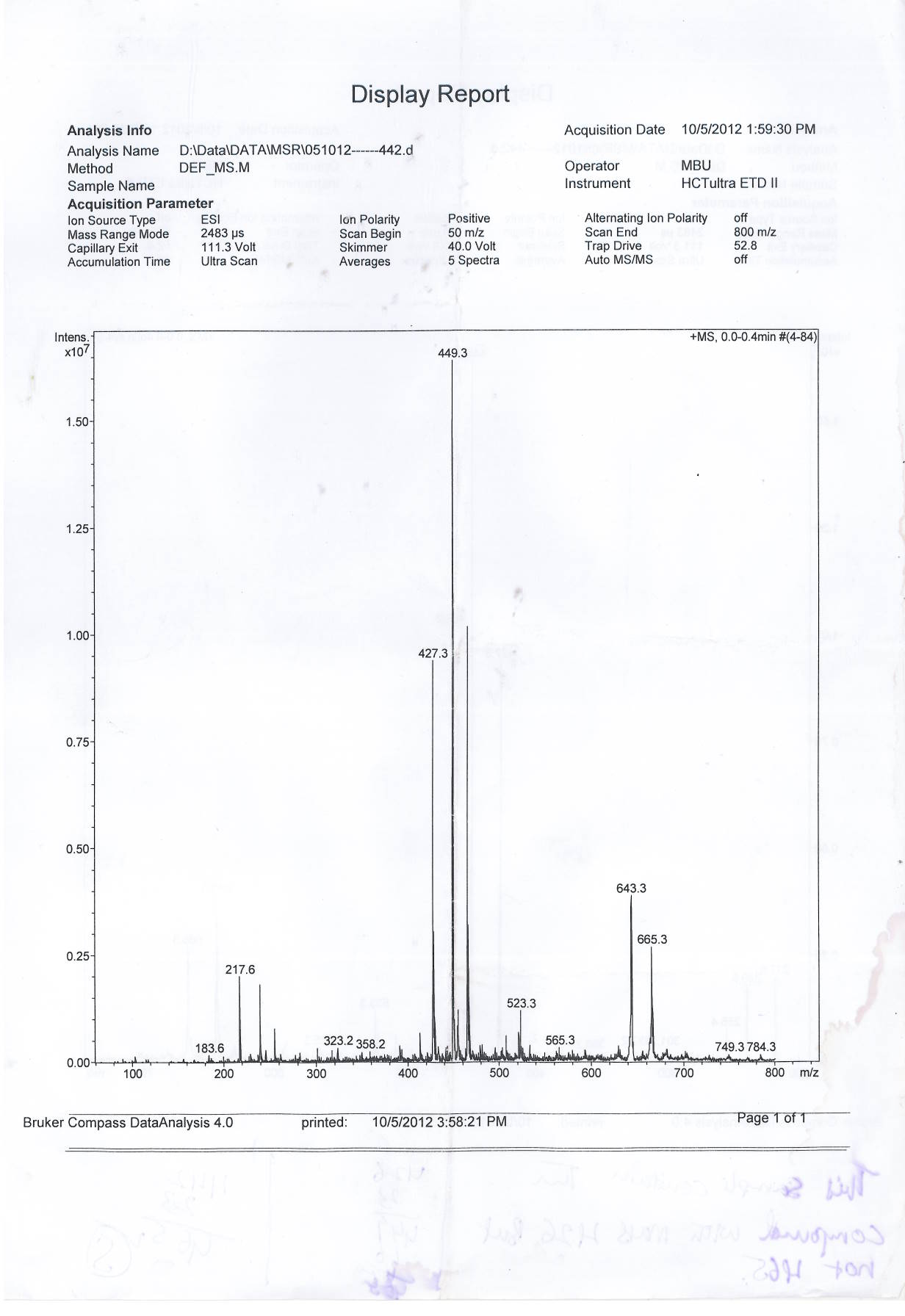


ESI-MS: 449.3 (426.2 + ^23^Na).

**8b**: 3,4,6,7-tetrahydro-3,3-dimethyl-9-phenyl-10-(pyridine-2-yl)-acridin-

1,8-(2*H*,5*H*,9*H*,10*H*)-dione


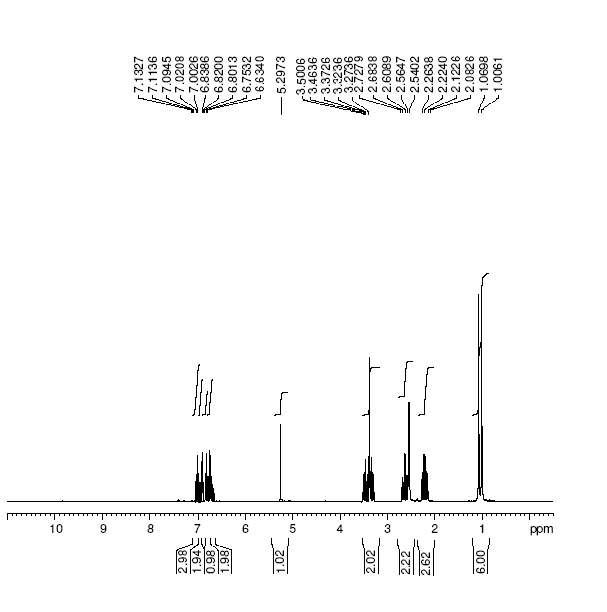


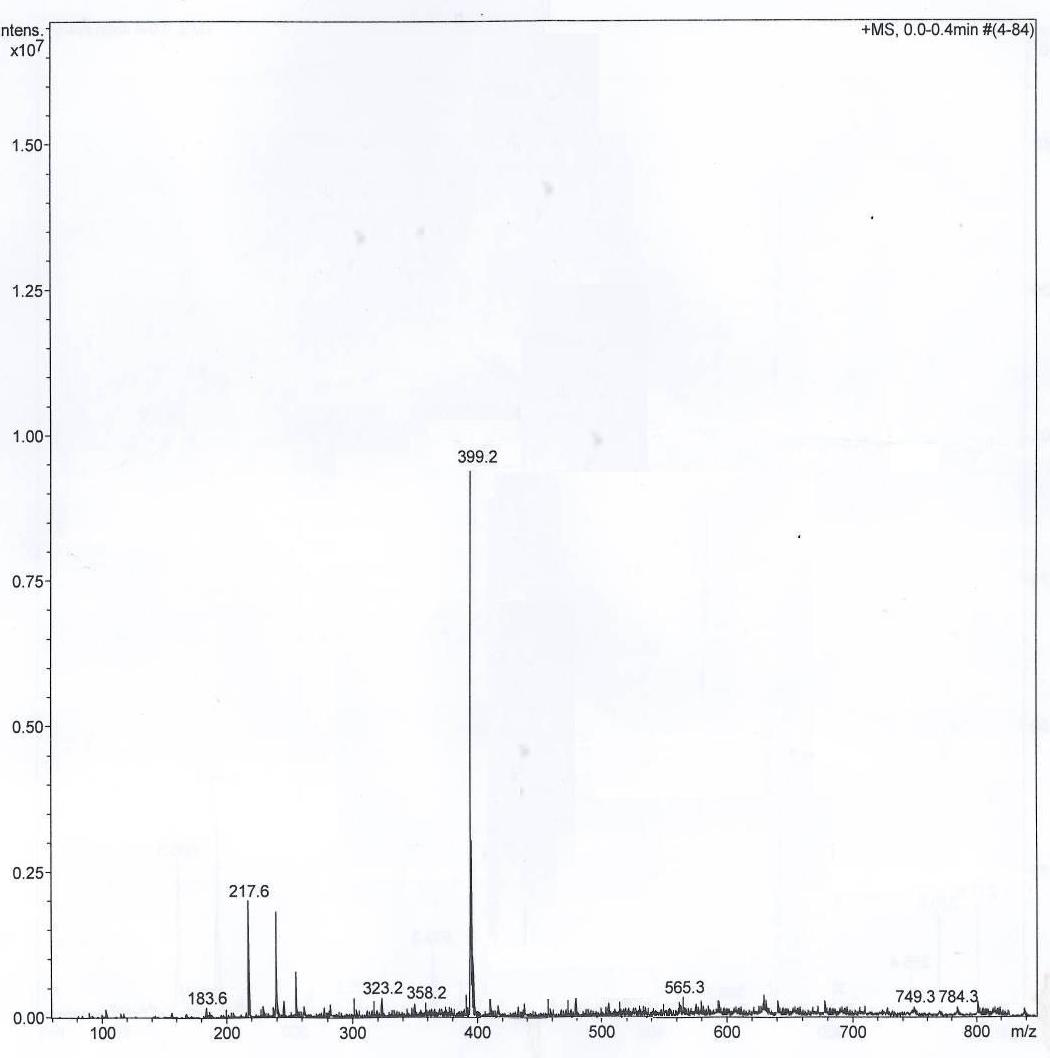


ESI-MS: 399.2 (398.2 + ^1^H)
